# EE-ACML: Energy-Efficient Adiabatic CMOS/MTJ Logic for CPA-Resistant IoT Devices †

**DOI:** 10.3390/s21227651

**Published:** 2021-11-18

**Authors:** Zachary Kahleifeh, Himanshu Thapliyal

**Affiliations:** 1 Department of Electrical and Computer Engineering, University of Kentucky, Lexington, KY 40506, USA; zachary.kahleifeh@uky.edu; 2 Department of Electrical Engineering and Computer Science, University of Tennessee, Knoxville, TN 37996, USA

**Keywords:** adiabatic logic, magnetic tunnel junction, correlation power analysis attack, side-channel attacks, low energy IoT, adiabatic clock generator

## Abstract

Internet of Things (IoT) devices have strict energy constraints as they often operate on a battery supply. The cryptographic operations within IoT devices consume substantial energy and are vulnerable to a class of hardware attacks known as side-channel attacks. To reduce the energy consumption and defend against side-channel attacks, we propose combining adiabatic logic and Magnetic Tunnel Junctions to form our novel Energy Efficient-Adiabatic CMOS/MTJ Logic (EE-ACML). EE-ACML is shown to be both low energy and secure when compared to existing CMOS/MTJ architectures. EE-ACML reduces dynamic energy consumption with adiabatic logic, while MTJs reduce the leakage power of a circuit. To show practical functionality and energy savings, we designed one round of PRESENT-80 with the proposed EE-ACML integrated with an adiabatic clock generator. The proposed EE-ACML-based PRESENT-80 showed energy savings of 67.24% at 25 MHz and 86.5% at 100 MHz when compared with a previously proposed CMOS/MTJ circuit. Furthermore, we performed a CPA attack on our proposed design, and the key was kept secret.

## 1. Introduction

Internet of Things (IoT) devices are necessary for the functions of modern life. IoT devices have a wide range of uses from the manufacturing sector [1] to everyday consumer products [2]. Many of these IoT devices are battery operated and thus reduced energy consumption is key to extending the use of these devices. Furthermore, many of these IoT devices, such as medical devices, transmit and store sensitive data thus making them prime targets for hardware attacks [3]. Flying ad hoc networks must be energy-efficient to remain mobile and functioning for long periods of time [4]. Further, the communication testbeds for these networks are a potential point for hardware attacks. One form of hardware attack IoT devices face is a side-channel attack. Side-channel attacks look to exploit secure information through a device’s side channels such as power consumption [5], timing [6], etc. Defense mechanisms against side-channel attacks can cause drastic energy increases; thus, the ideal solution should reduce energy consumption while defending against side-channel attacks [7,8].

**Figure 1:**
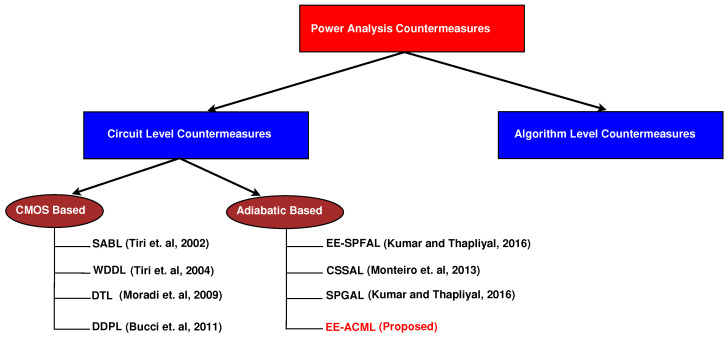
Correlation Power Analysis Countermeasures [7,8,9,11,12,13,14].

Novel design techniques such as adiabatic logic are promising to both reduce energy consumption and defend against a type of side-channel attack known as power analysis attacks [9]. Adiabatic logic reduces the dynamic energy consumption of a circuit by recycling stored charge in the load capacitor back into the clock to be used again [10]. Furthermore, dual-rail adiabatic circuits can be designed so that the circuits are balanced and power consumption remains uniform preventing information leakage [9]. Figure 1 shows the categories of countermeasures against Correlation Power Analysis Attacks (CPA).

Along with adiabatic logic, novel devices such as Magnetic Tunnel Junctions (MTJs) can also be used to design low energy and secure circuits [15]. MTJs are nonvolatile storage units that have low standby power, high integration density, and easy compatibility with CMOS [16,17,18]. MTJs can be added to CMOS structures to form nonvolatile ultra-low energy circuits [19].

In this article, we propose a novel hybrid adiabatic CMOS/MTJ logic named Energy-Efficient Adiabatic CMOS/MTJ Logic (EE-ACML). To demonstrate energy savings of EE-ACML integrated with an adiabatic clock generator, we designed one round of PRESENT. PRESENT is a lightweight encryption algorithm making it an ideal candidate for IoT devices. In our EE-ACML implementation of PRESENT, we showed that our circuit had energy savings of 67.24% at 25 MHz and 86.5% at 100 MHz when compared with a previously proposed CMOS/MTJ circuit. We have also shown that our proposed EE-ACML PRESENT implementation remains secure with the adiabatic clock generator implemented by performing a Correlation Power Analysis Attack (CPA) and determining the key was not revealed. A preliminary version of this paper appeared in [20].

This article is organized as follows: Section 2 discusses the necessary background information including adiabatic logic, power analysis attacks, MTJs, and CMOS/MTJ circuits. Section 3 discusses our proposed Energy-Efficient Adiabatic CMOS/MTJ Logic (EE-ACML) and our implementation of PRESENT. Section 4 discusses the simulation results of our proposed and comparison circuits. Section 5 discusses the CPA attack performed on the proposed circuit. Section 6 concludes the paper.

## 2. Background

In this section, we will cover the background information necessary to understand the proposed Energy-Efficient Adiabatic CMOS/MTJ Logic (EE-ACML). This section will discuss adiabatic logic, power analysis attacks, Magnetic Tunnel Junctions (MTJs), and adiabatic clock generators.

### 2.1. Adiabatic Logic and Power Analysis Attacks

**Figure 2:**
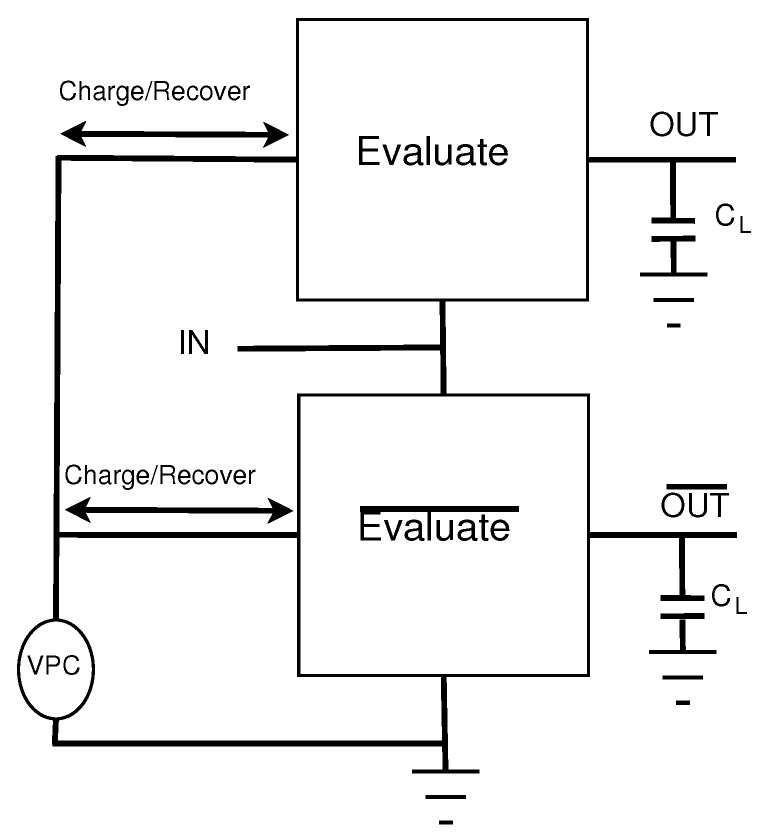
General Structure of Adiabatic Logic Circuits.

Adiabatic logic is an emerging design technique for designing low-energy circuits [10]. Adiabatic logic lowers energy consumption by recycling current stored within an adiabatic circuit’s load capacitor back into the clock. An adiabatic clock generator uses capacitors and inductors as storage elements for the recovered energy. The recovered energy is then reused in the next clock cycle thus reducing the energy of the circuit. The energy dissipated in an adiabatic circuit is given by:

(1) $$E_{diss}=\frac{RC}{T}CV_{dd}^{2}$$

where *T* is the period of the adiabatic clock, *C* is the capacitive load of the output, and $V_{dd}$ is the max voltage of the adiabatic clock, i.e., 1 V. By Equation (1), if the clock period T is greater than RC then the energy consumption will be lower than a standard CMOS circuit. Energy savings can be increased by increasing the period of the clock such that it is much greater than RC. Figure 2 illustrates the structure of an adiabatic circuit and its charging/discharging of the load capacitors.

**Figure 3:**
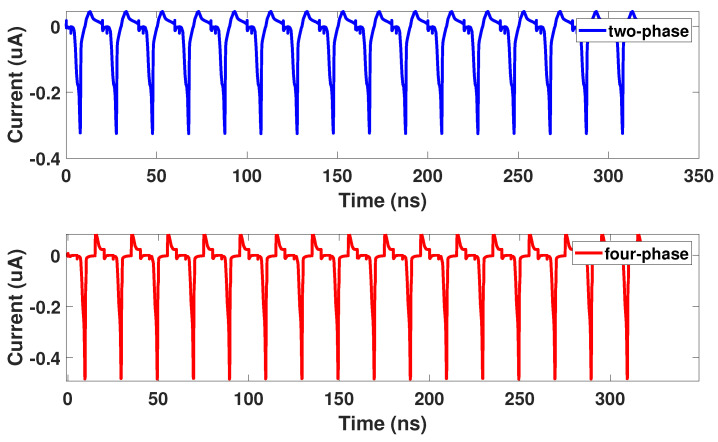
Uniform current consumption of an adiabatic logic gate.

Side-channel attacks attempt to steal information from a device’s inherent characteristics such as power consumption [5], timing [6], etc. In this article, we will focus on side-channel attacks in the form of power analysis attacks. Of the power analysis attacks, the Correlation Power Analysis Attack (CPA) is widely used because of its ability to target both symmetric and nonsymmetric cryptographic algorithms [21]. Different inputs of a circuit will result in different power consumption [5]. With this information, an attacker can measure hundreds of thousands of power profiles with controlled inputs to steal the secure encryption key. Masking and elimination are two methods to defend against power analysis attacks [22]. Masking aims to minimize correlation between data and power consumption such as in the proposed Bus-Invert Coding [23]. To defend against the CPA attack, we designed our circuits using a technique known as elimination [22]. Elimination aims to remove any variations in power consumption, so that each operation has uniform power consumption and thus no information leakage. An example of uniform power consumption can be seen in Figure 3, which shows the uniform current of a two and four-phase adiabatic gate.

### 2.2. Magnetic Tunnel Junctions and CMOS/MTJ Hybrid Circuits

**Figure 4:**
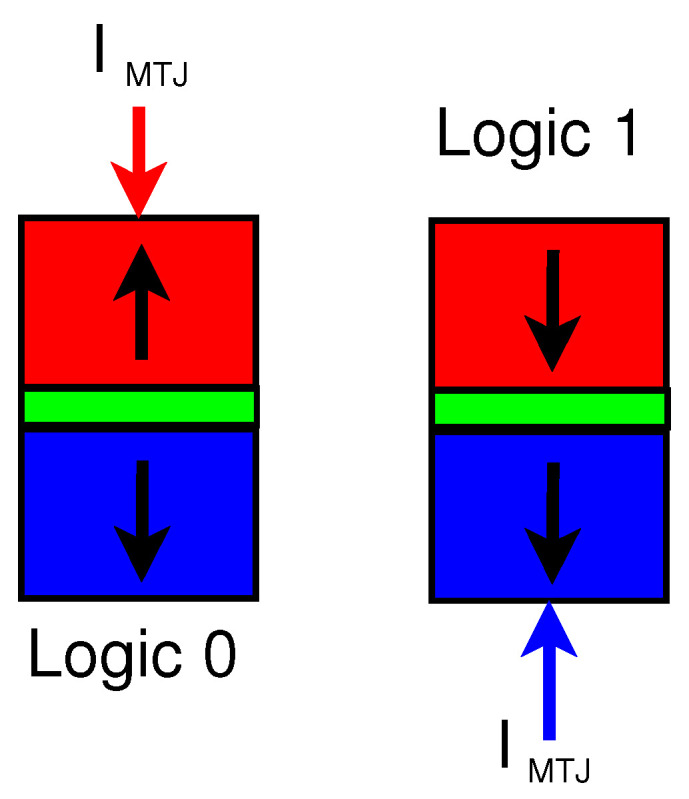
Structure of Magnetic Tunnel Junction (MTJ) with parallel and antiparallel states shown.

Magnetic Tunnel Junction (MTJ) is an emerging device that can be used to design low-energy and secure circuits. MTJs have numerous advantages such as ultra-low leakage power, high integration density, and easy compatibility with CMOS. The structure of the MTJ consists of two ferromagnetic (FM) layers and an oxide layer that acts as a barrier [24]. In most applications, one FM is layer is fixed while the other FM layer either takes a parallel orientation or an antiparallel orientation with respect to the fixed layer [25]. The structure of the MTJ device can be seen in Figure 4 with the bottom of the FM layer being fixed and the top FM layer has an antiparallel orientation for logic 0 or a parallel orientation for logic 1. The logic state of the MTJ is determined by the resistance of the device. A parallel magnetization ($R_{P}$) has lower resistance while an antiparallel magnetization ($R_{AP}$) has a higher resistance [26]. An important metric when discussing the reliability of an MTJ is the tunnel magnetoresistance ratio (TMR). The TMR is the difference in resistance between the two states and is defined as $TMR=\left(R_{AP}-R_{P}\right)/R_{P}$.

**Figure 5:**
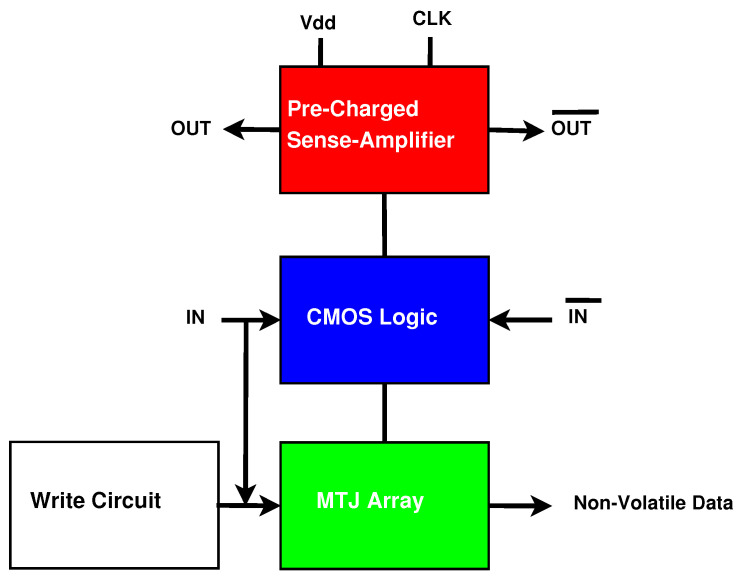
Generalized form of CMOS/MTJ circuits.

MTJ integration with CMOS structures has been implemented in previous work [16]. Figure 5 shows the generalized form of an existing version of a CMOS/MTJ circuit. The architecture contains the following components: a Pre-Charged Sense Amplifier (PCSA), a dual-rail CMOS logic tree, an MTJ array, and a writing circuit to switch the state of the MTJs when the inputs are changed. CMOS/MTJ circuits that switch frequently are not energy-efficient because of the substantial energy required to write to the MTJs [27].

**Figure 6:**
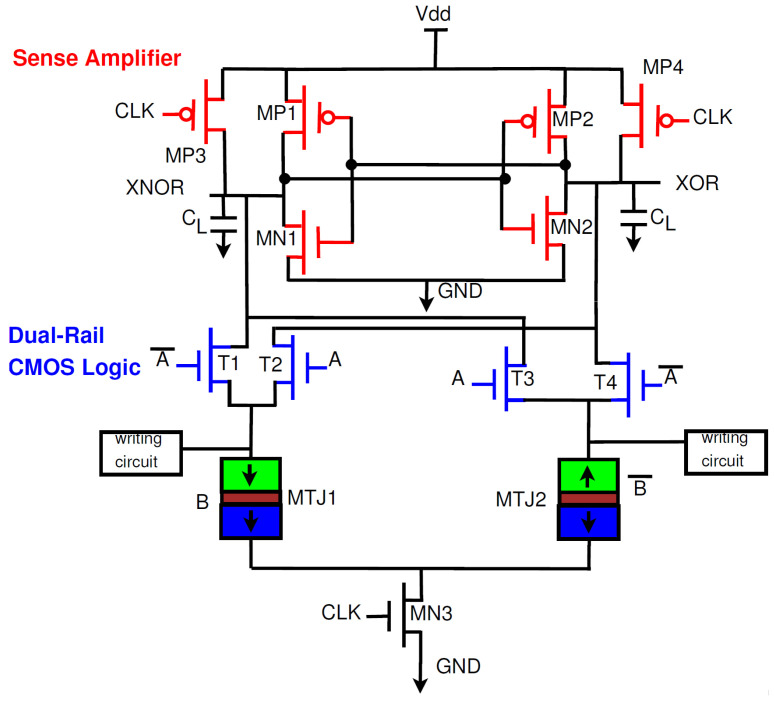
Hybrid CMOS-MTJ XOR circuit [16,28].

The operation of the PCSA can be explained through the existing PCSA-based CMOS/MTJ XOR gate (Figure 6) [16,28]. The PCSA has two stages depending on whether the clock is at logic 0 or logic 1. When CLK is at logic 0, MP3 and MP4 are both on and thus the outputs are pre-charged to logic 1. When CLK is at logic 1, MN3 is turned on and the outputs begin discharging to ground. One MTJ will be in the parallel state and the other MTJ will be in the antiparallel state, this results in a difference in resistance and thus the discharge speed will be faster through one MTJ. As an example, let us assume MTJ1 is in parallel mode and MTJ2 is in antiparallel mode. In this case, $R_{MTJ2}>R_{MTJ1}$ and as a result more current will flow through MTJ1 than in MTJ2. When the XOR node reaches the turn-on voltage of MP2, XNOR will be charged to logic 1 and XOR will be discharged to logic 0 through MN1.

### 2.3. Adiabatic Clock Generator

**Figure 7:**
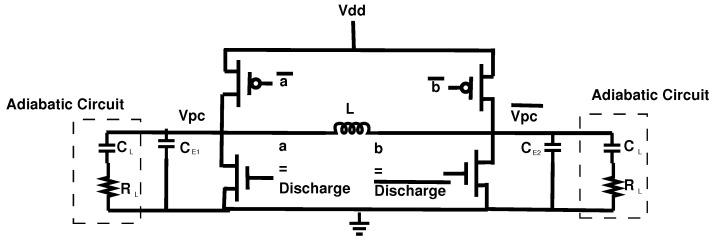
Structure of two-phase adiabatic clock generator [29].

This section will discuss the adiabatic Power Clock Generator (PCG) which is used to operate EE-ACML. The PCG used to operate our proposed circuit is shown in [29]. The PCG consists of an external inductor and the load of the adiabatic circuit resulting in an RLC resonant circuit. The structure of the two-phase clock generator is shown in Figure 7. The PCG structure contains two PMOS and two NMOS transistors with four control signals.

**Figure 8:**
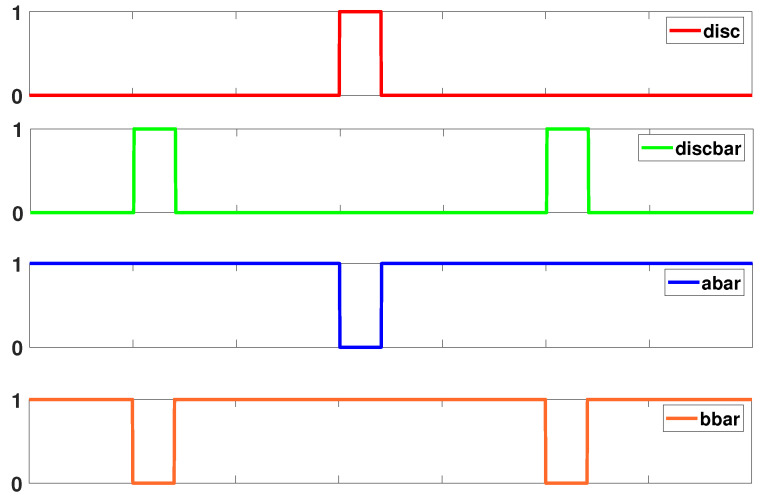
Signals used to control operation of Power Clock Generator (PCG).

EE-ACML and many other CPA resistant adiabatic circuits rely on discharge signals to defend against power analysis attacks. Discharge signals are used to ensure both outputs have no remaining charge before the next cycle begins. The discharge signals are placed when their respective clock signals are at GND. The discharge signals play an important role in lowering the correlation between the power and logic operation. Thus, in a previous work we developed a novel way for discharge and $\bar{discharge}$ to have a dual-function: (i) Control signals for the clock generator (ii) discharge the load capacitors of the adiabatic logic circuit [30]. This duality allows for a reduced number of external signals and simpler designs. The timing diagram of the external control signals is shown in Figure 8.

### 2.4. Security Parameters for CPA Resistant Circuits

We will use two parameters to evaluate the security of our proposed design, Normalized Energy Deviation and Normalized Standard Deviation. The first parameter Normalized Energy Deviation (NED) is defined as

(2) $$NED=\left(E_{max}-E_{min}\right)/E_{max}$$

NED is the normalized difference between the minimum and maximum energy consumption within a set of possible energy consumption per bit transition. Normalized Standard Deviation (NSD), is defined as

(3) $$NSD=\frac{\sigma_{e}}{\bar{E}}$$

where $\sigma_{e}$ is the standard deviation of the energy dissipated by the circuit per input transition, and $\bar{E}$ is the average energy dissipation. The NSD tells us the standard deviation of each energy value from the average energy. Lower NED and NSD values indicate less variation in power consumption and thus less information leakage.

## 3. Proposed Energy-Efficient Adiabatic CMOS/MTJ Logic (EE-ACML)

**Figure 9:**
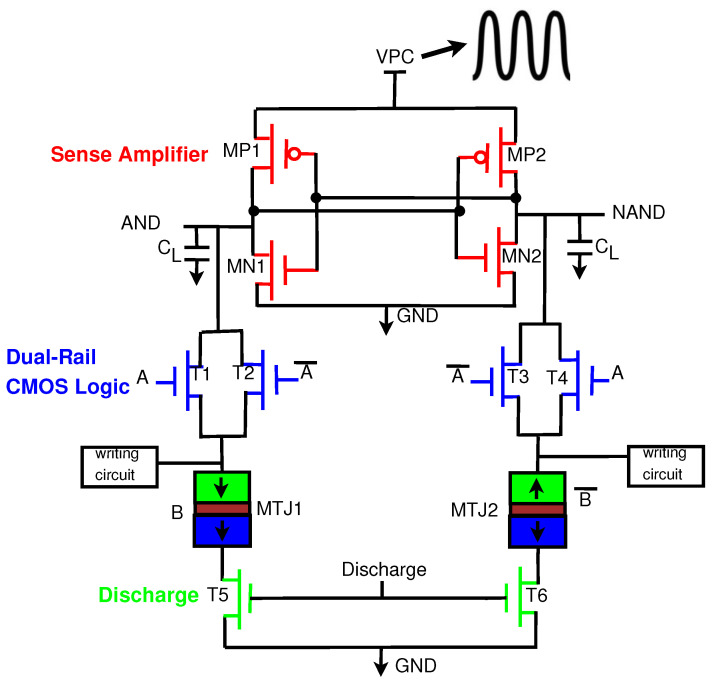
Proposed Energy-Efficient Adiabatic CMOS/MTJ AND/NAND gate.

**Figure 10:**
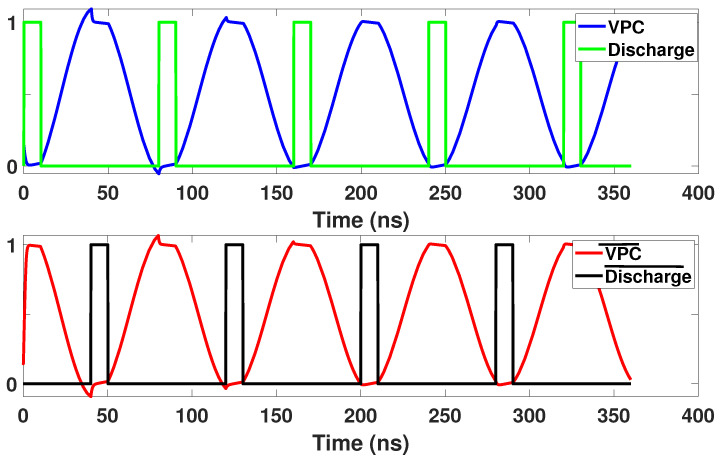
CPA-resistant two-phase adiabatic logic clocking scheme used in EE-ACML [30].

This section introduces the generic structure of our proposed Energy-Efficient Adiabatic CMOS/MTJ Logic (EE-ACML) and its operation. The proposed AND/NAND gate circuit can be seen in Figure 9. We can see that the structure consists of an adiabatic clock connected to a 2P2N Sense Amplifier. T1-T4 make up the NMOS only evaluation network connected to two MTJs (MTJ1 and MTJ2) with parallel and antiparallel configurations. Finally, transistors T5 and T6 are used to discharge any current stored in the load capacitors at the end of a clock cycle (When VPC is 0). A single EE-ACML gate requires two signals to operate correctly, a two-phase adiabatic clock and a discharge signal. When more than two gates are cascaded together, EE-ACML requires two sinusoidal clocks 180${}^{\circ }$ out of phase as well as two discharge signals in phase with the respective clocks. The complete adiabatic clocking waveform used to operate EE-ACML is shown in Figure 10.

### 3.1. Proposed Adiabatic CMOS/MTJ Operation

This section will explain the operation of EE-ACML. The operation will be explained with the AND/NAND gate seen in Figure 9.

#### 3.1.1. Discharge Stage

**Figure 11:**
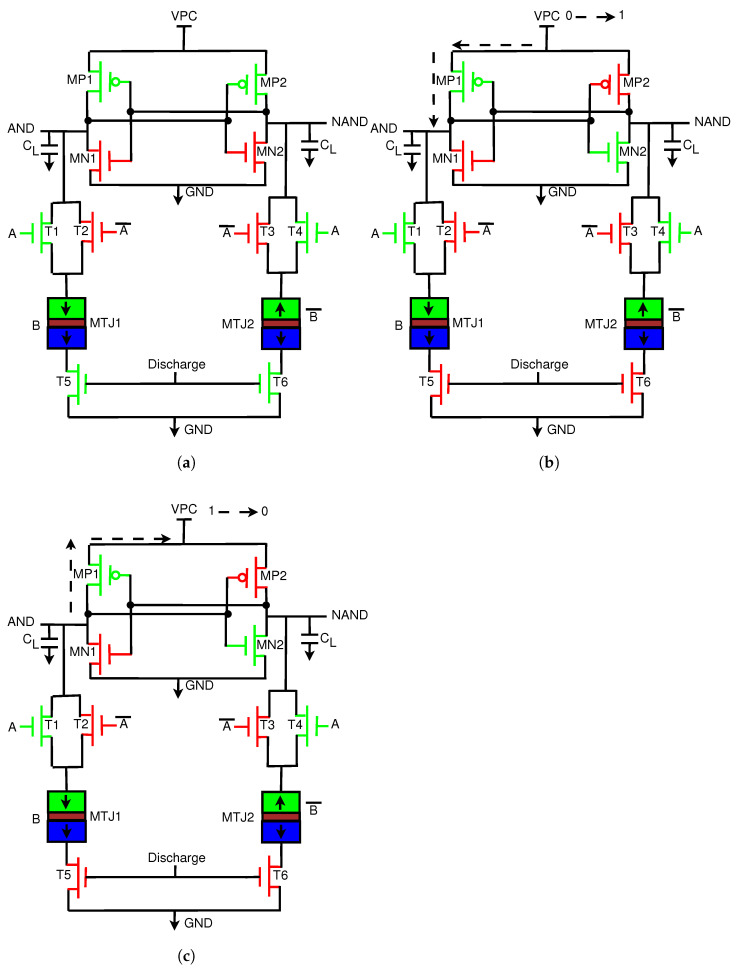
Operation of the proposed Energy-Efficient Adiabatic CMOS/MTJ AND/NAND gate. (**a**) Discharge stage of operation; discharge = 1, VPC = 0, A = 1, B = 1. (**b**) Evaluation phase of operation; VPC = 0 -> 1, discharge = 0, A = B = 1. (**c**) Recovery phase of operation; VPC = 1 -> 0, discharge = 0, A = B = 1.

At the start, we assume that A = 1, MTJ1/B = 1, discharge = 1, and VPC = 0. The operation is illustrated in Figure 11a. When the discharge signal is 1, T5 and T6 are on, and MP2 is connected to ground through T1 and T5. When MP2 is on, AND follows VPC, which is currently 0. When AND is at 0, MP1 is also turned on, and NAND is also at 0.

#### 3.1.2. Evaluation Phase

In this phase, the inputs remain at their current values. Discharge is now 0, and VPC begins to rise from 0 to 1. The operation of this stage is illustrated in Figure 11b. AND and NAND both rise with VPC; however, due to the difference in resistance between MTJ1 and MTJ2, one path will conduct more current. In this case, MTJ1 has lower resistance, and thus more current will flow through MP1. This will cause MP2 to turn off and MN2 to turn on. AND will rise with VPC to its peak value, while NAND will pull down to logic 0 through MN2.

#### 3.1.3. Recover Phase

The operation of this stage is illustrated in Figure 11c. In this phase, VPC begins to drop from VDD to GND. At this point, AND is at VDD and thus has a higher potential than VPC. Current will begin to travel from the high potential node to the low potential node at VPC. Current is stored in the inductors and capacitors that make up the clock to be reused again in the next cycle, and thus energy is recovered. At the end of the phase, the discharge signal will go to VDD to remove any remaining charge in the load capacitors.

### 3.2. Low Energy and Secure EE-ACML PRESENT Implementation

To show the energy efficiency and security of our proposed EE-ACML, we use the lightweight block cipher PRESENT as a case study [31]. Battery-operated IoT devices have tight energy and area constraints; thus, the lightweight PRESENT is an ideal choice for these devices. In this article, we demonstrate the energy efficiency and security of our proposed design using the 80-bit version of PRESENT. PRESENT has 31 rounds and consists of three stages: add round key, substitution layer, and permutation layer. Here, we design one round to demonstrate energy efficiency and security.

#### 3.2.1. Substitution Box

**Figure 12:**
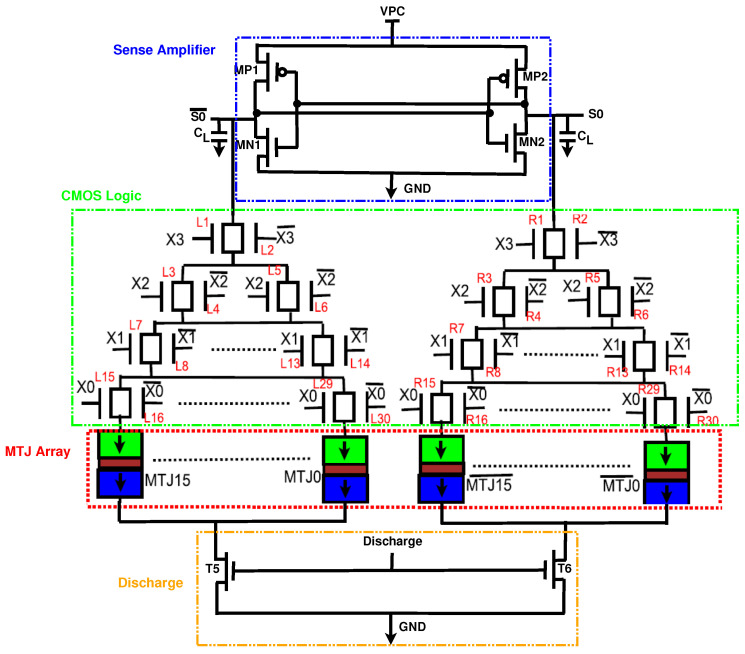
Proposed EE-ACML Look-Up-Table (LUT).

One of the components of PRESENT is the substitution box (S-box), which performs a nonlinear substitution. When implemented with CMOS, the S-box is prone to Correlation Power Analysis Attacks (CPA). Thus, we implemented the S-box with the proposed EE-ACML. In applications where data switch frequently, the energy consumption of MTJ-based circuits is high as a result of the write energy [27]. With this in mind, we designed our S-box using a Look-Up-Table (LUT)-based structure, so we only had to write to the MTJs once. The structure of the proposed S-box is shown in Figure 12. The MTJs contains the outputs to the S-box, which are constant, and thus do not need to be switched [31].

#### 3.2.2. Add Round Key (XOR) Layer

**Figure 13:**
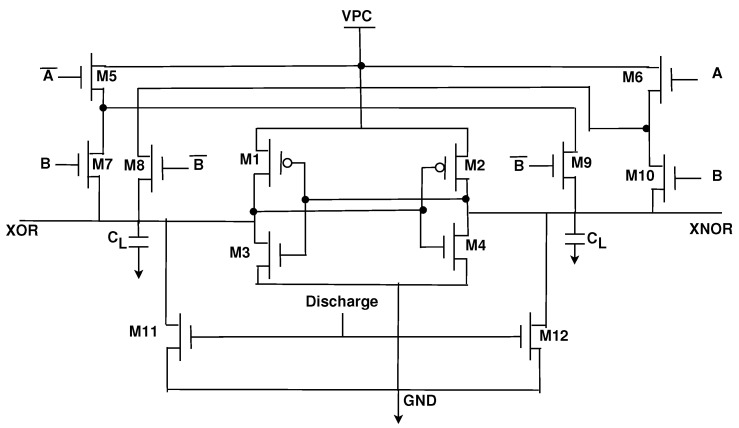
2-EE-SPFAL XOR Gate used to implement the add round key stage of PRESENT [30].

Another component of PRESENT is the add round key layer, which consists of an array of XOR gates. The CMOS/MTJ implementation of PRESENT utilizes a CMOS/MTJ-based XOR gate and thus cannot switch data often unless it pays a large energy penalty. In our implementation, we designed our XOR gate using 2-EE-SPFAL [32]. 2-EE-SPFAL is a recently proposed two-phase CPA resistant adiabatic circuit. The two-phase clocking scheme allows for 2-EE-SPFAL to work in tandem with EE-ACML. Utilizing the 2-EE-SPFAL XOR gate means we can switch data frequently without having to worry about high energy consumption. The 2-EE-SPFAL XOR gate can be seen in Figure 13.

## 4. Results

This section presents the results of EE-ACML with the clock generator implemented. Simulations were performed using Cadence Spectre simulator with 45nm standard CMOS technology. We designed our circuits such that the MTJ switching was at a minimum; thus, we modeled our MTJs using a resistor. The resistance was determined by the models provided in [33] and the parameters shown in Table 1.

### 4.1. Analysis of the Energy-Efficiency of the Proposed EE-ACML with Integrated Power Clock Generator

In this section, we examine the effect the adiabatic power clock generator has on EE-ACML. In our first study, we examined the effects of change in frequency and inductor on energy per cycle. In this analysis, the capacitor was kept constant while the inductor was changed based on Equation (4).

(4) $$f=\frac{1}{2\pi \sqrt{L\frac{C}{2}}}$$

**Figure 14:**
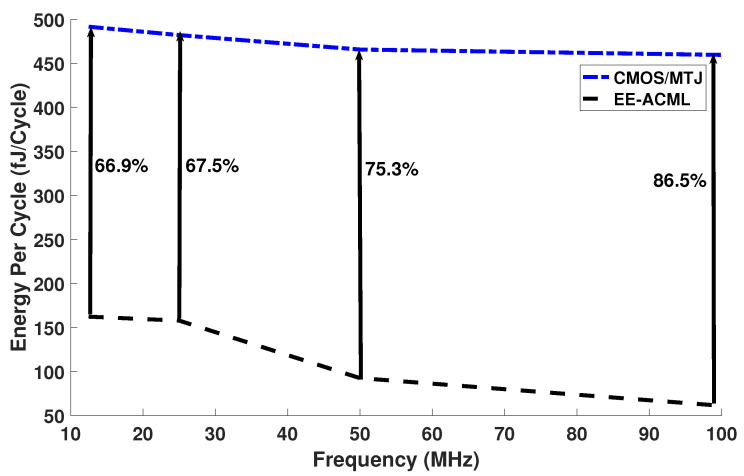
Energy per cycle comparison between proposed EE-ACML and CMOS/MTJ.

The capacitor and inductor values used in our simulations are shown in Table 2. The results of our analysis can be seen in Figure 14 and in Table 3. At 25 MHz and a capacitor and inductor value of 351.67 fF and 230.49 μH, our proposed circuit consumed 157.81 fJ/Cycle, while the CMOS/MTJ implementation consumed 482.0 fJ/Cycle. This resulted in 67.25% energy savings between the two implementations of PRESENT. At 100 MHz and an inductor value of 14.40 μH, our proposed circuit consumed 459.56 fJ/Cycle, which resulted in energy savings of 86.58%.

In our next study, we kept a constant frequency and varied the capacitor and inductor values to determine the effect on energy per cycle. Different values of inductors and capacitors resulted in varying power consumption of the RLC clock generator, which can be seen in Equation (5). Equation (5) gives the power consumption of a resonant RLC circuit in which *L* and $\omega_{0}$ vary with inductance and capacitance.

(5) $$P_{avg}=\frac{V^{2}R\omega^{2}}{R^{2}\omega^{2}+L^{2}{\left(\omega^{2}-\omega_{0}^{2}\right)}^{2}}$$

**Figure 15:**
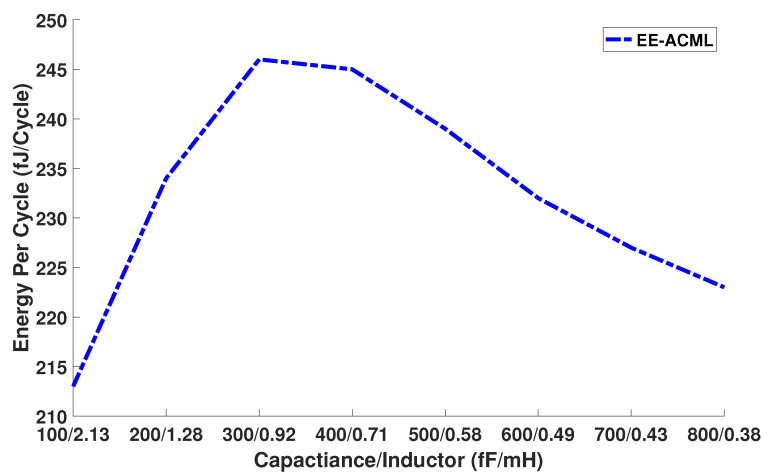
Effect of different inductor and capacitor values on energy consumption.

Thus, we theorize that the energy per cycle trend seen in Figure 15 is a result of the changing capacitors and inductors and thus the power of the RLC circuit.

**Figure 16:**
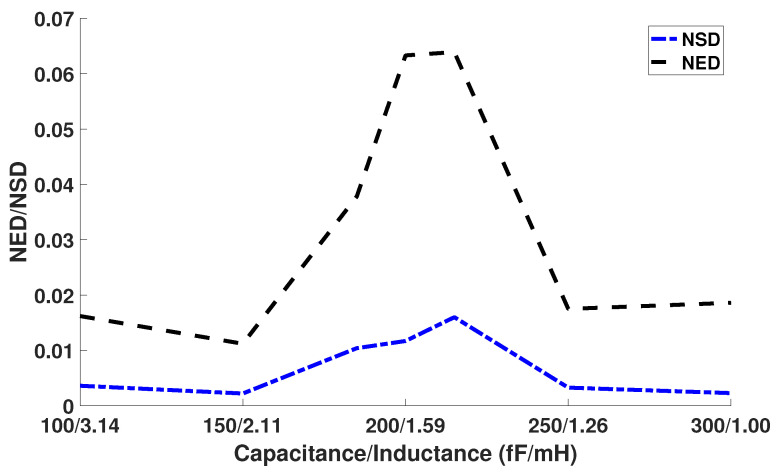
Effect of changing capacitor and inductor on NED and NSD.

The adiabatic clock generator can also affect the security of our adiabatic CMOS/MTJ circuit. We varied the inductor and capacitor to determine the effect it has on Normalized Energy Deviation and Normalized Standard Deviation. The results can be seen in Figure 16. From Figure 16, we can see that the NED and NSD values peak at certain inductor and capacitor values. We theorize that this is a result of the RLC power clock generator having higher power consumption at these inductor and capacitor values thus causing more variation in overall power consumption. We conclude that there is a certain capacitor and inductor value that will result in a more robust countermeasure against CPA attacks.

### 4.2. Device Count of Proposed Energy-Efficient Adiabatic CMOS/MTJ Logic

The area consumption is an important metric when designing integrated circuits for IoT devices; thus, in this section, we will present the device count of EE-ACML.

Table 4 shows the device count for various CMOS, CMOS/MTJ, and EE-ACML circuits. We can see that the EE-ACML AND/NAND gate has one less transistor than the CMOS/MTJ-based AND/NAND gate. The CMOS/MTJ substitution box has 4 extra transistors when compared to the EE-ACML substitution box.

We also recorded the number of transistors for one round of PRESENT. The CMOS/MTJ implementation of PRESENT has 4 fewer transistors than the EE-ACML implementation. This is because the CMOS/MTJ implementation uses the CMOS/MTJ XOR/XNOR gate while the EE-ACML implementation uses the 2-EE-SPFAL-based XOR/XNOR gate, which has more transistors. The tradeoff of using the MTJ-based XOR/XNOR gate is it cannot be switched frequently without consuming substantial energy. EE-ACML uses fewer transistors than the CMOS implementation of PRESENT. This is because Flip-Flops are added to each CMOS output to synchronize the outputs.

### 4.3. Analysis of Security of the Proposed EE-ACML S-Box

In this article, we simulate and record the energy numbers of the PRESENT substitution box in order to calculate the Normalized Energy Deviation (NED) and Normalized Standard Deviation (NSD) values. Our simulations and results are with the adiabatic clock generator implemented. Table 5 shows the NED and NSD values for EE-ACML as well as a CMOS/MTJ S-box [16] and a purely adiabatic circuit 2-Energy Efficient-Secure Positive Feedback Adiabatic Logic (2-EE-SPFAL) [30]. From Table 5 we can see that our proposed adiabatic CMOS/MTJ circuit consumes average energy of 41.6 fJ, while the CMOS/MTJ implementation consumes 78.2 fJ, and the 2-EE-SPFAL circuit consumes 35.2fJ at 12.5 MHz. Furthermore, our proposed S-box has a NED value of 0.0011 and an NSD value of 0.002, both lower than the CMOS/MTJ and 2-EE-SPFAL implementation of the PRESENT S-box.

## 5. Correlation Power Analysis Attack on EE-ACML-Based PRESENT

In this section, we will demonstrate EE-ACML-based PRESENT resilience against a CPA attack. The adiabatic clock generator was implemented again to determine if the circuit remained secure. As the key is used for the operation of the substitution box, it was used as the attack point. The CPA attack was performed by following the steps described in [34]. The simulation was performed at 12.5 MHz with a key value of 2 ${\left(0010\right)}_{b}$. In the field, CPA attacks usually require hundreds of thousands of traces to steal encryption keys as a result of electrical noise and other nonideal factors. However, in our simulations we required fewer traces, because the noise factors were not present. To demonstrate the ability of our CPA attack, we performed one on a CMOS-based PRESENT circuit and determined that the key could be stolen [20]. We used the same CPA attack on the EE-ACML-based PRESENT to confirm the CPA-resistant ability of EE-ACML.

**Figure 17:**
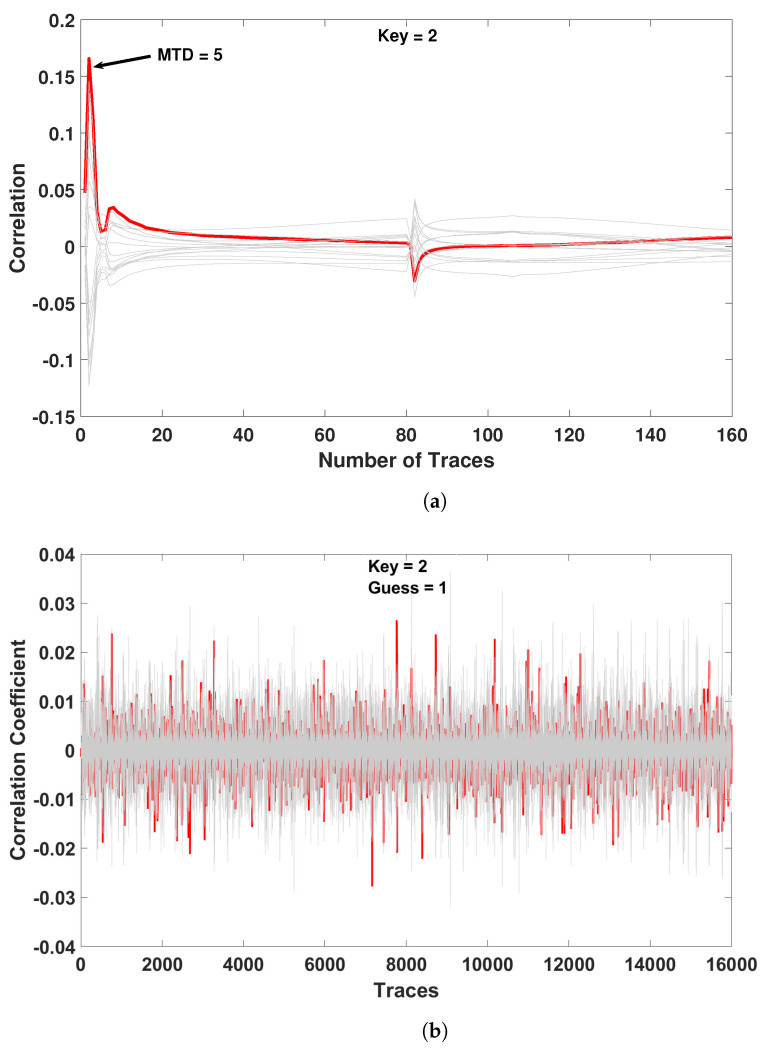
Correlation power analysis performed on EE-ACML implementation of PRESENT-80. (**a**) Successful CPA attack on CMOS-based implementation of PRESENT S-box with key = 2. (**b**) Unsuccessful CPA attack on EE-ACML-based implementation of PRESENT S-box with key = 2.

In our attack on the CMOS-based PRESENT, we utilized 160 traces and were able to steal the encryption key. Figure 17a shows a successful CPA attack against the CMOS implemented PRESENT S-box for a key value of 2. The Measurements to Disclosure (MTD) was five traces. In our attack on the EE-ACML-based PRESENT we used 16,000 traces and were unable to retrieve the key. Figure 17b shows an unsuccessful attack when the key value is 2, where the attack produced a guess of 1. The unsuccessful CPA attack on EE-ACML-based PRESENT shows it is a promising solution to defending against power analysis attacks on IoT devices.

## 6. Discussion and Conclusions

In this article, an adiabatic CMOS/MTJ architecture known as Energy-Efficient Adiabatic CMOS/MTJ Logic (EE-ACML) was presented and shown to be both energy efficient and secure. An adiabatic clock generator was implemented to show energy savings, security, and reliability remained. The novel circuit provided substantial energy savings when compared to a CMOS/MTJ circuit found in the literature [16]. As a case study, we constructed one round of PRESENT and showed our circuit remained energy efficient. Our circuit consumed 156.81 fJ/Cycle, which amounts to 67.25% energy savings when compared to the CMOS/MTJ implementation. To demonstrate secure operation we performed a Correlation Power Analysis attack on our EE-ACML-based PRESENT circuit and showed that the key remained secret.

Our work demonstrates the effectiveness of both adiabatic logic and magnetic tunnel junctions in designing low-energy and secure circuits. The low energy consumption makes the novel circuits ideal candidates to be implemented within battery-constrained IoT devices. The implementation of an adiabatic clock generator also aids in proving our proposed circuits’ ability to remain energy efficient and secure. To further scrutinize the security of our device, machine learning-based CPA attacks can be performed on our design to determine the resilience [35]. Machine learning-based CPA attacks require fewer traces and higher test accuracy.

## Figures and Tables

**Table 1:** Magnetic Tunnel Junction parameters used in simulations.

Parameter	Description	Value
$t_{sl}$	Thickness of free layer	1.3 nm
a	Length of surface long axis	40 nm
b	Width of surface short axis	40 nm
$t_{ox}$	Thickness of the Oxide barrier	0.85 nm
TMR	Tunnel Magneto Resistance ratio	150%
RA	Resistance Area Product	5 $\Omega \mu^{2}$
Area	MTJ layout surface	40 nm × 40 nm × π/4
$R_{p}$	Parallel resistance	6.21 kΩ
$R_{ap}$	Antiparallel resistance	18.64 kΩ

**Table 2:** One round of PRESENT inductor and capacitor values at various frequencies.

Frequency	Capacitor (fF)	Inductor (μH)
12.5 M	351.67	921.96
25 M	351.67	230.49
50 M	351.67	57.62
100 M	351.67	14.40

**Table 3:** One round of PRESENT energy per cycle (fJ/Cycle) of EE-ACML and CMOS/MTJ [16].

Frequency	Proposed EE-ACML	CMOS/MTJ [16]	Energy Savings (%)
12.5 M	162.25	491.56	66.99
25 M	157.81	482.00	67.25
50 M	114.71	465.62	75.36
100 M	61.19	459.56	86.58

**Table 4:** Device counts of various CMOS, CMOS/MTJ, and EE-ACML-based circuits.

Logic Family	Logic Gate	Transistor Count
EE-ACML	NAND	10
	XOR	10
	SBOX	264
	1-Round PRESENT	4996
CMOS/MTJ [16]	NAND	11
	XOR	11
	SBOX	268
	1-Round PRESENT	4992
CMOS	NAND	4
	XOR	8
	SBOX	216
	1-Round PRESENT	5120

**Table 5:** Normalized Energy Deviation and Normalized Standard Deviation values for EE-ACML-based S-box.

Parameter	Proposed EE-ACML	CMOS/MTJ [16]	2-EE-SPFAL [30]
$E_{min}\left(fJ\right)$	41.4	77.3	34.2
$E_{max}\left(fJ\right)$	41.9	79.1	36.3
$E_{avg}\left(fJ\right)$	41.6	78.2	35.2
NED	0.011	0.022	0.056
NSD	0.002	0.006	0.012

## Data Availability

Not Applicable.
